# Way to Kill Mosquitoes

**Published:** 1898-09

**Authors:** 


### WAY TO KILL MOSQUITOES.

Two and one-half hours are required for a mosquito to develop from its first stage, a speck resembling cholera bacteria, to its active and venomous maturity. The insect in all its phases may be instantly killed by contact with minute quantities of permanganate of potassium. It is claimed that one part of this substance in fifteen hundred of solution distributed in mosquito marshes will render the development of lavæ impossible; that a handful of permanganate will oxidize a ten-acre swamp, kill its embryo insects, and keep it free from organic matter for thirty days at a cost of twenty-five cents; that with care a whole State may be kept free of insect pests at a small cost. An efficacious method is to scatter a few crystals widely apart. A single pinch of permanganate has killed all the germs in a thousand-gallon tank.—*The Public Health Journal.*

